# Storage Stability and In Vitro Bioaccessibility of Microencapsulated Tomato (*Solanum Lycopersicum* L.) Pomace Extract

**DOI:** 10.3390/bioengineering9070311

**Published:** 2022-07-13

**Authors:** Luiz C. Corrêa-Filho, Diana I. Santos, Luísa Brito, Margarida Moldão-Martins, Vítor D. Alves

**Affiliations:** LEAF—Linking Landscape, Environment, Agriculture and Food, Associated Laboratory TERRA, Instituto Superior de Agronomia, Universidade de Lisboa, Tapada da Ajuda, 1349-017 Lisboa, Portugal; lucaalbernaz@gmail.com (L.C.C.-F.); dianaisasantos@isa.ulisboa.pt (D.I.S.); lbrito@isa.ulisboa.pt (L.B.); mmoldao@isa.ulisboa.pt (M.M.-M.)

**Keywords:** agro-industry, microencapsulation, bioactive compounds, in vitro digestion, yogurt

## Abstract

Tomato pomace is rich in carotenoids (mainly lycopene), which are related to important bioactive properties. In general, carotenoids are known to react easily under environmental conditions, which may create a barrier in producing stable functional components for food. This work intended to evaluate the storage stability and in vitro release of lycopene from encapsulated tomato pomace extract, and its bioaccessibility when encapsulates were incorporated in yogurt. Microencapsulation assays were carried out with tomato pomace extract as the core material and arabic gum or inulin (10 and 20 wt%) as wall materials by spray drying (160 and 200 °C). The storage stability results indicate that lycopene degradation was highly influenced by the presence of oxygen and light, even when encapsulated. In vitro release studies revealed that 63% of encapsulated lycopene was released from the arabic gum particles in simulated gastric fluid, whereas for the inulin particles, the release was only around 13%. The feed composition with 20% inulin showed the best protective ability and the one that enabled releasing the bioactives preferentially in the intestine. The bioaccessibility of the microencapsulated lycopene added to yogurt increased during simulated gastrointestinal digestion as compared to the microencapsulated lycopene alone. We anticipate a high potential for the inulin microparticles containing lycopene to be used in functional food formulations.

## 1. Introduction

Worldwide, large quantities of fruit and vegetable wastes (e.g., seeds, skins, and pomaces) are discarded annually due to processing and poor storage facilities. As these food wastes rich in organic compounds are easily susceptible to microbiological deterioration, they cannot be introduced into the food chain without some processing. However, organic by-products can be valorized by extracting the bioactive compounds present in their composition for subsequent use as functional ingredients for food, pharmaceutical and cosmetic products, as they are considered to have beneficial effects on health [1,2,3].

Changes in modern lifestyle and the growing awareness of the link between diet and health, as well as new processing technologies, have led to an increased interest on the development of new healthy food products. Among them, a lot of attention has been driven to the development of functional food products enriched with natural functional bioactive molecules. Many bioactive compounds, such as carotenoids, extracted from natural sources have been shown to possess several biological activities, such as antimicrobial, antibacterial, antifungal, antiviral, anti-inflammatory, anti-obesity, anticholesteremic, antihypertensive, and antioxidant functions [4].

Carotenoids such as lycopene and β-carotene are antioxidants naturally present in some food products, and may be recovered as concentrated extracts from agroindustrial by-products, such as tomato pomace [2,4,5]. Tomato and tomato-based foods are the main sources of lycopene and are considered important contributors of carotenoids to the human diet [6]. However, the same properties that make carotenoids useful in healthy tissue function create challenges in preventing the degradation of carotenoids in food products due to the presence of unsaturated bonds in molecular structure [7,8]. As such, carotenoids are compounds very instable to processing and storage and their stability is affected by pH, exposure to light, temperature, oxygen, and acid media. These factors may influence the biological activity, nutritional quality, and the development of undesired flavors and of food. Therefore, the stability is an important aspect to consider for use of carotenoids as antioxidants and colorants in foods [9,10,11].

Microencapsulation of bioactive compounds, such as carotenoids and anthocyanins, is a process that enables the transformation of a liquid mixture with bioactives into a powder, which facilitates their handling, transport, storage, dosage, and application [12]. Furthermore, the encapsulation process has been extensively used for the stabilization of the bioactive compounds, improving their bioaccessibility and the control of their release rate in targeted sites [12,13,14].

Spray drying has been widely used in the food processing field due to its ease of industrialization, continuous production, and low cost, and it produces dry particles of good quality and with low water activity. During the spray drying process, evaporation of the water from the wall material occurs rapidly which maintains the core temperature below 100 °C, despite the use of high temperatures in the process. Therefore, this is an advantageous method for the encapsulation of heat sensitive compounds [13,15].

The characteristics of the ideal protective (wall) material depends on its intended purpose. Composition of the wall material influences the efficiency of the bioactive (core) material protection and its controlled release. Generally, wall materials should produce low viscosity solutions or suspensions at high solids content, emulsifying properties (especially for hydrophobic core materials), and resistance to the gastrointestinal tract [16].

Arabic gum is a polysaccharide complex derived from acacia trees. In general, arabic gum imparts low viscosity in aqueous media, presents high solubility in water, presents good emulsifying properties and good volatile retention [15,17]. Another interesting wall material used in the food industry is inulin due to its nutritional benefits and diversified technical and functional properties. Inulin is a fructooligosaccharide (FOS) commercially obtained from chicory root and it is composed of fructose units with β (2-1) bonds with glucose at the end of the chain [18]. Inulin is a dietary fiber that improves calcium bioavailability and it has prebiotic effects as it is degraded by certain probiotic bacteria present in the colon such as bifidobacteria and lactobacilos [19].

There is an interest in designing microencapsulates to release the encapsulated bioactive compounds in the intestine, where they may be absorbed into the blood stream. The literature indicates that the various types of wall materials used for the encapsulation of bioactive compounds influence differently on the bioacessibility/bioavailability of their compounds. In vivo digestion methods generally provide more reliable results but are time consuming and expensive. Alternatively, in vitro digestion models are useful methods that have been used in the first stages of the study [16,20].

The valorization of tomato pomace with the extraction of lycopene-rich fractions, and subsequent stabilization by spray drying using various wall materials (e.g., polysaccharides and proteins) has been studied [21,22]. However, detailed studies are still needed, as many factors beyond the type of wall material influence the spray drying encapsulation process and the properties of encapsulates obtained, such as the range of air inlet temperature used and the wall material concentration. In a previous work, a comprehensive study of such factors was carried out using Arabic gum and inulin as wall materials [8]. As such, the aim of this work was to go further, and study the stability during storage of the encapsulated lycopene-rich extract from tomato pomace. Furthermore, the in vitro release rate of lycopene in simulated gastric and intestinal fluids was evaluated, both from microparticles alone and from microparticles incorporated in yogurt with probiotic bacteria. In the latter case, beyond lycopene bioaccessibility, the effect of inulin particles on the survival of probiotic bacteria was also studied.

## 2. Materials and Methods

### 2.1. Materials

Tomato pomace (*Solanum lycopersicum* L.) was supplied by the HIT Group (Marateca, Portugal). Arabic gum (LabChem, Zelienople, PA, USA) and Inulin (Alfa Aesar, Kandel, Germany) were used to form the protective matrix. Commercial probiotic yogurt (Actimel, Danone, Paris, France) and Natural liquid yogurt (Drink, Milbona, Neckarsulm, Germany) were purchased from a local supermarket to be used as the food matrix.

2,2′-Azinobis (3-ethylbenzothiazoline-6-sulphonic acid) diammonium salt (ABTS), lycopene, β-carotene, pepsin from porcine gastric mucosa (P6887), lipase from porcine pancreas (L3126) and pancreatin from porcine pancreas (P7545) were purchased from Sigma–Aldrich (Steinheim, Germany). 6-hydroxy-2,5,7,8-tetramethylchroman-2-carboxylic acid (Trolox) was obtained from Acrós Organics (Geel, Belgium). Potassium persulfate (K_2_S_2_O_8_), potassium chloride (KCl), calcium chloride (CaCl_2_), sodium hydrogen carbonate (NaHCO_3_) and ethanol were purchased from Panreac AppliChem. Acetonitrile, dichloromethane, ethyl acetate and sodium chloride (NaCl), were obtained from Honeywell (Seelze, Germany). MRS broth was purchased from Biokar diagnostics (Beavais, France).

### 2.2. Tomato Pomace Extract Preparation

Frozen tomato pomace comprised of skin and seeds was lyophilized in a Lyo Quest freeze-dryer at −47 °C and 0.100 mbar for 48 h, powdered in a cutting mill and finally classified in a system of sieves arranged in columns in order to separate particles with a diameter of less than 0.5 mm. The tomato powder was stored under vacuum and protected from light.

The extraction of bioactive compounds (mostly lycopene and β-carotene) was carried out in a soxhlet apparatus, in which 15 g of tomato pomace powder was extracted with 300 mL of ethanol until the solvent became completely colorless after contact with the powder (around 5 h). After extraction, the solvent was evaporated by rotary vacuum evaporator (Rotavapor^®^ R II BUCHI) and the extract obtained was placed in amber glass flasks and stored at −20 °C until analysis.

### 2.3. Preparation of Emulsions

Arabic gum was dissolved in distilled water under stirring overnight at room temperature, while inulin was dissolved in distilled hot water (70 °C). Two wall material concentrations (10 and 20% *w/w*) were used for encapsulation of tomato pomace extract. After the full hydration of the polymer molecules, concentrated tomato pomace extract (15% *w*/*w*, polymer basis) was added into each polymer solution and emulsions were produced by stirring with an Ultra-Turrax T25 (IKA, Staufen im Breisgau, Germany) at 13,500 rpm for 1 min at ambient temperature. A volume of 75 mL of emulsion was prepared for each experimental condition.

### 2.4. Spray Drying Conditions

The resultant solutions were fed at a rate of 3.7 mL·min^−1^ to a co-current spray dryer (Lab-Plant SD-05, Huddersfield, UK) equipped with a 0.5 mm diameter nozzle. The feed solution was kept under magnetic stirring and the pressure of the compressed air set at 1.7 bar. The inlet temperature was 160 and 200 °C, for inulin and arabic gum assays, respectively. The dried powders obtained were collected and stored under vacuum protected from light.

### 2.5. Morphological Characterization of Microparticles

The morphology of the particles obtained by spray drying was observed by Scanning Electron Microscopy (SEM). Samples were coated with a mixture of gold (80%) and palladium (20%) in a vacuum chamber, and analyzed using a Hitachi S2400 scanning microscope (Scotia, NY, USA) operated at 10 kV with a magnification of 1000×.

### 2.6. Analytical Methods

To assess its antioxidant activity and the concentrations of lycopene and β-carotene, the concentrated extract was diluted into a 1:1 acetonitrile:dichloromethane (ACN:DCM) solution 0.1% (*m/v*). All analytical measurements were carried out in triplicate.

#### 2.6.1. Carotenoids Content in the Tomato Pomace Extract

The concentration of lycopene and β-carotene in the tomato pomace extract was quantified by HPLC using an UltiMate-3000 HPLC system (Dionex, Sunnyvale, CA, USA) equipped with a column oven at 30 °C. Carotenoid separation, identification, and quantification was performed using a reversed phase C_18_ 5 µm, 120 Å (4.6 × 150 mm) column by a gradient elution of acetonitrile:water:tetraethylammonioum (900:100:1) as solvent A and ethyl acetate as solvent B. The elution started with a mixture of 75% solvent A and 25% solvent B. After 10.50 min the solvent A was decrease to 59 % and after 20.0 min to 0%. At 21.0 min the solvent A returned to the initial condition (75%), remaining constant up to 31 min. The flow rate was 1 mL.min^−1^ and the running time was 31 min. The injection volume of the samples was 20 µL. The identification of carotenoids was based on their retention time of a peak compared with the carotenoids’ standards. Calibration curves were carried out using standard lycopene and β-carotene with different concentrations (1–10 mg/L) using 1:1 ACN:DCM solution as solvent. Detection was carried out with a DAD-3000 diode-array detector with a wavelength of 475 and 472 nm for lycopene and 440 nm for β-carotene. The chromatogram obtained is presented in the supplementary file (Figure S1). The amount of β-carotene and lycopene in the samples was expressed as mg/g_extract_.

#### 2.6.2. Antioxidant Activity of the Tomato Pomace Extract

The total antioxidant activity of samples was performed by radical scavenging activity assessment expressed as Trolox Equivalent Antioxidant Activity (TEAC). An ABTS stock solution was prepared by dissolving ABTS in water at a 7 mM concentration. The ABTS^+^ solution was produced by reaction of 5 mL of ABTS stock solution and 88 µL of a 140 mM potassium persulfate (K_2_S_2_O_8_) solution to give a final concentration of 2.45 mm. This solution was kept in a dark room at room temperature for 12–16 h. Before analysis, the ABTS^+^ solution was diluted with ethanol to obtain an initial absorbance value of 0.700 ± 0.05 at 734 nm.

For the evaluation of the antioxidant activity of tomato pomace extract itself, a volume of 30 µL of diluted extract with ACN:DCM solution was mixed with 3000 µL of ABTS^+^ solution, followed by incubation for 6 min in the dark. Then, the absorbance was measured in a spectrophotometer (Unicam, UV/Vis Spectrometer–UV4) at a wavelength of 734 nm. A calibration curve was performed using Trolox, as standard antioxidant, at the concentration range of 250–2000 µM in ethanol.

#### 2.6.3. Loading Capacity

For the determination of the concentration of the bioactive compounds present in the microparticles a mass of 20 mg of microparticles was added to 10 mL of a mixture of ACN and DCM (1:1). The suspension was homogenized with an Ultra-Turrax T25 (IKA, Staufen, Germany) at 13,500 rpm during 3 min, in order to break the microparticles. After mixing, the suspension was placed in amber glass flasks and kept away from light for about 12 h at 5 °C. Afterwards, the suspension was filtered with a syringe filter (Nylon 25 mm diameter, pore size of 0.45 µm; Fisher Scientific, Hampton, NH, USA) into an HPLC vial. The concentration of bioactive compounds in the liquid phase was quantified by HPLC, in the same way used for the tomato pomace extract. The loading capacity (LC) of the particles was expressed as the mass of carotenoids per mass of particles.

#### 2.6.4. Antioxidant Activity of the Encapsulated Material

For the measurement of the antioxidant activity (AA) of the encapsulated molecules, the microparticles core material were previously extracted with of a 1:1 acetonitrile:dichloromethane (ACN:DCM) as described in the previous section. Afterwards, a volume of 800 µL of supernatant was mixed with 2200 µL of ABTS^+^ solution, followed by the steps described in Section 2.6.1 for the fresh extract.

### 2.7. Storage Stability of Microencapsulated Tomato Pomace Extract

Samples of microparticles loaded with tomato pomace extract, and free lycopene powder, were placed in desiccators under four conditions as follows: (1) Dark + O_2_; (2) Dark + N_2_; (3) Light + O_2_; and (4) Light + N_2_. The relative humidity and temperature were kept at 33% and 25 °C, respectively, during all the experiment. Microparticles were periodically analyzed for 27 days to access the stability of encapsulated bioactives to oxygen and light, compared to that of free lycopene.

Lycopene loss was determined as the difference between the original and the obtained content of lycopene at different storage times. The quantification of carotenoids and antioxidant activity of bioactives in microparticles was performed in triplicate, employing the same methodology described previously.

### 2.8. In Vitro Digestion Studies

#### 2.8.1. Microencapsulated Tomato Pomace Extract

In vitro digestion studies of microencapsulated tomato pomace extract were performed under static conditions in simulated gastric (SGF, pH 1.7) and intestinal (SIF, pH 6.5) fluids without enzymes as described by Gomes, et al. [21]. These studies were conducted in both fluids in parallel. The compositions of the simulated gastric and intestinal fluids are shown on Table 1. The pH of the simulated digestive fluids was adjusted with 1 M HCl or 1 M NaOH solutions. The release studies were carried out in a shaking thermal water bath (type 3047, Kotterman, Germany) with controlled temperature at 37 °C. In each release essay, 50 mg of microparticles was suspended in 5 mL of simulated fluids, in 15 mL Falcon tubes, under mild stirring for 2 h.

#### 2.8.2. Yogurt with Incorporated Microparticles

In vitro digestion studies of the food matrix, yogurt, enriched with microparticles were performed using simulated gastric fluid (SGF, pH 1.7) and intestinal fluid (SIF, pH 6.5) as described in the previous section with some modifications. Enzymes were added to the simulated gastric and duodenal fluids (Table 1). Porcine pepsin and lipase from porcine pancreas were added to achieve 2000 U·mL^−1^ and 40 U·mL^−1^, respectively, in the gastric digestion mixture. Pancreatin was added to achieve 100 U·mL^−1^ in the intestinal digestion mixture. A mass of 2.5 g of yogurt was placed in a Falcon tube containing 50 mg of each type of microparticles. The food matrix release assay was performed sequentially. First, the mass of 2.5 mg of yogurt with microparticles was suspended in 2.5 mL of simulated gastric fluid in 15 mL Falcon tubes under mild stirring for 2 h. Subsequently, 5.0 mL of simulated duodenal fluid was added in the same tube and let under mild shaking for more 2 h.

#### 2.8.3. Carotenoid Bioaccessibility

The bioaccessibility of carotenoids in the microparticles was determined during the in vitro digestion process in order to obtain their release profile. At regular time intervals, different tubes were taken from the thermal bath, centrifuged at 8000 rpm for 20 min at 10 °C to separate the microparticles, recovering the supernatant (micelle fraction) containing the released bioactive compounds. The bioaccessibility of carotenoids in yogurt was determined only at the end of each stage of digestion (gastric and intestinal).

For the extraction of carotenoids from both the microparticles alone and from the food matrix with microparticles, 3 mL of chloroform was added in the tubes containing the raw digesta (before centrifugation) or micelle fraction, vortexed, and then centrifuged at 4000 rpm for 10 min at 10 °C. The organic layer was collected and quantified by HPLC. The bioaccessibility of carotenoids was calculated as the ratio of the carotenoids concentration present in the micelle fraction by the carotenoids concentration in the raw digesta.

### 2.9. Viability of Bacteria from the Probiotic Yogurt (Actimel^®^)

Survival of the bacteria contained in the probiotic yogurt with and without microparticles was evaluated during the in vitro digestion studies. At regular time intervals (0, 2, 4, 6, and 8 h), different Falcon tubes were taken from thermal bath to analyze the survival of bacteria present in the yogurt. An aliquot of 0.1 mL of the sample contained in each Falcon tube was diluted in 0.9 mL of ringer solution and then 0.1 mL of that diluted sample was spread on MRS (de Man, Rogosa and Sharp) agar plate. The microbial count was made after incubation at 30 °C for 72 h. The results were expressed as CFU·mL^−1^ of yogurt.

MRS broth was prepared by dissolving 55.3 g MRS and 20 g agar-agar into 1 L of hot distilled water. The broth was autoclaved at 121 °C for 15 min, cooled down to 50 °C in a water bath, and then poured into the petri dishes. The media was used within 2 days.

### 2.10. Statistical Analysis

The experimental data were statistically evaluated using Statistica^TM^ v.8 Software (StatSoft Inc., Tulsa, OK, USA, 2007). Statistically significant differences (*p* < 0.05) between samples were evaluated using Tukey test.

## 3. Results and Discussion

### 3.1. Characterization of Tomato Pomace Extract

The concentration of lycopene and β-carotene in the tomato pomace extract was 15.19 ± 0.42 and 0.63 ± 0.02 mg·g^−1^ extract, respectively, which corresponds to 6.26 mg lycopene·g^−1^ dry tomato pomace and 0.26 mg β-carotene·g^−1^ dry tomato pomace. In the assessment of antioxidant activity, the extract possessed 536 ± 4.75 µmol trolox·g^−1^ extract that is equivalent to 35.27 ± 1.25 µmol trolox·mg^−1^ lycopene. Lower values of lycopene content (0.143 ± 0.004 mg·g^−1^ tomato concentrate) and antioxidant activity (11.3 ± 0.6 μmol Trolox·g^−1^ tomato concentrate) in tomato concentrate obtained by reverse osmosis followed by lower diafiltration were found by Souza, et al. [23]. This difference observed in the values of the lycopene content and antioxidant activity can be explained by the different way of obtaining the extract and the source of the raw material. The lycopene content of the pomace is dependent on the type of tomato plant cultivar.

### 3.2. Storage Stability of Microencapsulated Tomato Pomace Extract

It is well-known that the storage conditions, i.e., the pH, temperature, light, oxygen, and water activity, are important factors for preserving sensitive materials such as bioactives, flavors, and microorganisms. The stability of microparticles is defined as a state in which their physical (particle size, shape, size diameter), chemical and microbial characteristics are maintained unchanged throughout the storage period [15]. The non-microencapsulated and microencapsulated tomato pomace extracts were stored under different environmental conditions to evaluate the effect of the wall material on the protection of lycopene and antioxidant activity.

#### 3.2.1. Lycopene Content

**Figure 1:**
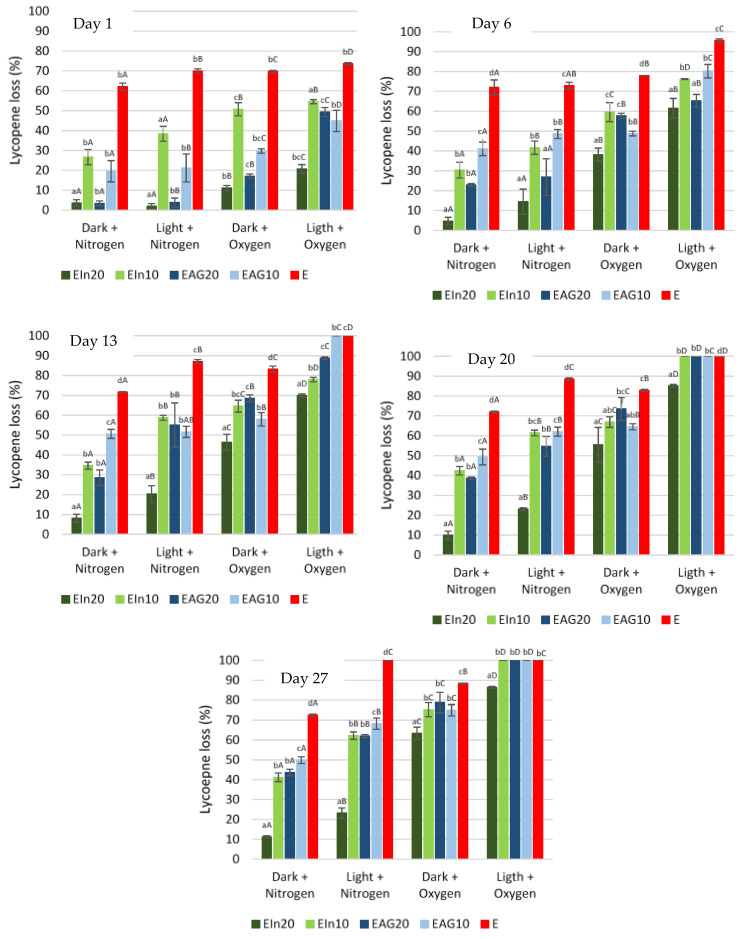
Lycopene loss over the storage period under different conditions. Different letters represent statistically significant differences (Tukey test *p* < 0.05): uppercase letters (effect of environmental conditions on lycopene loss); lowercase letters (effect of samples on lycopene loss). EIn20–20% Inulin; EIn10–10% Inulin; EAG20–20% Arabic gum; EAG10–10% Arabic gum; E–tomato pomace extract.

Figure 1 represents the lycopene loss during storage for each environmental conditions and samples studied.

The loading capacity obtained for each wall material formulation was evaluated at the beginning of storage time (day 0). The loading capacity values obtained for 20 and 10% wall material were 1.1 and 1.3 mg lycopene·g^−1^ particles for inulin, and 0.9 and 1.5 mg lycopene·g^−1^ particles for arabic gum, respectively. The lycopene content at the start of the storage time was considered to be 100% to exclude the differences in the amount of lycopene obtained between formulations of the wall materials.

Overall, both inulin and arabic gum microcapsules with tomato pomace extract presented improved stability during storage compared to the free extract, showing the importance of microencapsulation in retarding the degradation of the bioactive compounds. Nevertheless, the loss of lycopene and antioxidant activity increased with increasing storage time in all storage conditions.

Similar results were reported in studies on microencapsulation of carotenoids by spray drying. Souza, Hidalgo-Chávez, Pontes, Gomes, Cabral, and Tonon [23] microencapsulated carotenoids from tomato concentrate into different types of wall materials (maltodextrin, whey protein isolate and Capsul^®^) and Álvarez-Henao, et al. [24] studied the stability of lutein-loaded microcapsules using maltodextrin, arabic gum, and modified starch as wall materials. Çam, et al. [25] also found the same behavior in phenolic compounds when they evaluated the stability of phenolics from microencapsulated pomegranate seeds under storage at 5 °C for 3 months.

The encapsulated samples of tomato pomace extract with 20 and 10% wall material presented a lycopene degradation of 11.2 and 41.2% for inulin, and 43.6 and 49.9% for arabic gum, after 27 days of storage in dark and nitrogen. On the other hand, when stored under adverse environmental conditions (presence of light and oxygen), all the samples showed a complete degradation of the lycopene, except the particles with 20% inulin that presented a lycopene degradation of 86.4%. Overall, the results indicate that lycopene degradation was highly influenced by the presence of oxygen and light, demonstrating that the bioactive compounds should be preserved in absence of these conditions, even when encapsulated with the studied wall materials.

In terms of wall material concentration, a greater protection of lycopene was observed for the highest concentration of both wall material (20%). According to Bhandari, et al. [26], higher solids content results in greater protection of microencapsulated material and thus, greater stability. As mentioned, all samples of microencapsulated tomato pomace extract showed a better lycopene retention under less adverse environmental conditions (dark and nitrogen), presenting a degradation of less than 50%, whereas the non-microencapsulated extract was higher than 70%. Furthermore, under these conditions, the microparticles with 20% of inulin were the more efficient ones in protecting the bioactives, with a lycopene degradation of 11.2%.

Other researchers also studied carotenoid degradation in microparticles produced by spray drying using different type of wall materials. Souza, Hidalgo-Chávez, Pontes, Gomes, Cabral, and Tonon [23] stored lycopene-loaded microcapsules from tomato concentrate produced by spray drying in the dark for 28 days at 25 °C. Maltodextrin, whey protein isolate and Capsul^®^ were used as wall materials. After the storage period, a loss of lycopene between 67 and 93% was observed. Rocha et al. [27] used Capsul^®^ as wall material to produce lycopene-loaded microparticles. They observed that the particles stored at 25 °C in vacuum and away from the light had lycopene degradation of 41.32% after 73 days. Matioli and Rodriguez-Amaya [28] evaluated the effect of light on the degradation of lycopene from red-fleshed guava pulp microencapsulated in arabic gum during storage. The authors observed that more than 50% of encapsulated lycopene was degraded on day 19 and day 7 when stored in light and dark, respectively, both at room temperature. Álvarez-Henao, Saavedra, Medina, Jiménez Cartagena, Alzate and Londoño-Londoño [24] evaluated the storage stability of lutein-loaded microparticles produced by spray drying using arabic gum and the mixture of maltodextrin, arabic gum and modified starch (1:1:1) as wall materials. The samples were stored at 40 °C and relative humidity of 75%. At the end of 20 days of storage under the specified conditions, the microcapsules with arabic gum and the mixture of different wall materials showed a degradation in lutein content of 79 and 65%, respectively, while the degradation in non-encapsulated lutein extract was 89.5%. According to the authors, the film forming ability of the arabic gum used for producing the microparticles was not enough to provide stability over storage time, which was also observed in this work for both concentrations of arabic gum studied. However, this capacity increased with the addition of the modified starch and maltodextrin in the wall material formulation, which according to the authors can be due to the amylose present in the starch.

In terms of the different storage conditions studied, in the presence of light and oxygen, the stability of the lycopene contained within the microparticles was more negatively affected by the presence of oxygen. The best storage condition was Dark + Nitrogen, followed by Light + Nitrogen. The conditions containing oxygen, Dark + Oxygen followed by Light + Oxygen, were the two worst for storage of the microcapsules. Pelissari, et al. [29] reported that the degradation of lycopene was highly influenced by the presence of oxygen and storage temperature when evaluating the storage stability of microcapsules produced by spray chilling using shortening composed of hydrogenated and interesterified cottonseed, soy, and palm oils, and arabic gum as wall materials. According to Rodriguez-Amaya [30], a loss of carotenoids during storage occurs mainly due to non-enzymatic oxidation, since the carotenoid molecules are highly unsaturated and easily prone to oxidative degradation. This degradation depends on the availability of oxygen and the structure of the carotenoid being stimulated by the presence of light and heat.

#### 3.2.2. Antioxidant Activity

**Figure 2:**
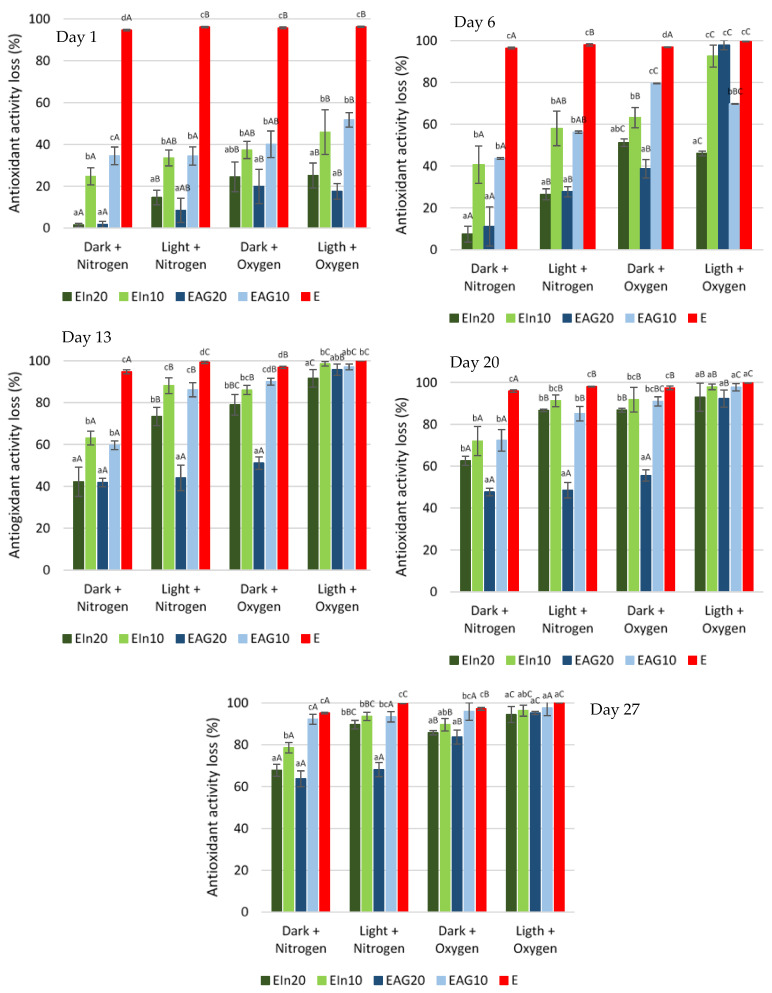
Antioxidant activity loss over the storage period under different conditions. Different letters represent statistically significant differences (Tukey test *p* < 0.05): uppercase letters (effect of environmental conditions on antioxidant activity loss); lowercase letters (effect of samples on antioxidant activity loss). EIn20–20% Inulin; EIn10–10% Inulin; EAG20–20% Arabic gum; EAG10–10% Arabic gum; E-tomato pomace extract.

The antioxidant activity (AA) values obtained for each wall material formulation was evaluated at the beginning of storage time (day 0). The antioxidant activity values obtained for microcapsules with 20 and 10% of wall material were 10.7 and 14.0 µmol Trolox·mg^−1^ lycopene for inulin, and 22.2 and 11.6 µmol Trolox·mg^−1^ lycopene for arabic gum, respectively. Additionally, the AA value of non-encapsulated extract was 35.3 µmol Trolox·mg^−1^ lycopene. The AA at the start of the storage time was considered to be 100% to exclude the differences in the amount of antioxidant capacity obtained between formulations of the wall materials. Figure 2 represents the AA loss during storage for each environmental conditions and samples studied.

The encapsulated samples of tomato pomace extract with 20 and 10% of wall material showed a loss of antioxidant activity of 67.7 and 78.5% for inulin and 63.8 and 92.2% for arabic gum, after 27 days of storage under dark and nitrogen. On the other hand, when stored under the presence of light and oxygen, all samples had a high loss of antioxidant activity, above 94%. Still, from the 13th day of storage, 91% of the antioxidant activity had already been decreased, even in microencapsulated bioactive compounds. As was observed in lycopene degradation, the loss of antioxidant activity was influenced by the presence of oxygen and light as well as the type and concentration of the wall material used. Both inulin and arabic gum at 20% were significantly more able to maintain a higher antioxidant capacity than the other samples analyzed.

Gomes, et al. [31] evaluated the stability of microcapsules of arabic gum and maltodextrin loaded with lycopene-rich watermelon juice during storage for 15 days at room temperature. The results showed that the loss of AA was proportional to the loss of the lycopene content until the fifth day of storage. From this point, a greater loss of antioxidant activity was observed compared to loss of lycopene. At the end of storage time, the amount of lycopene in the particles reduced by 41.9%, while the antioxidant capacity reduced by 91.2%. According to the authors, first, the loss of the antioxidant activity was related to the degradation of the carotenoids. Afterwards, the most pronounced loss of antioxidant capacity after the fifth day was due to the continuous formation of cis isomers and other low antioxidant compounds produced by heating during the atomization process. Ramakrishna, et al. [32] studied the effect of light and temperature on the storage stability of microcapsules loaded with tamarillo juice rich in carotenoids and anthocyanins. Maltodextrin, arabic gum and modified starch were used as the wall material during the spray drying process. At the end of 28 days of storage in the presence of light, the loss of antioxidant activity varied between 6.59 and 9.85%. Meanwhile, at the end of 84 days of storage at 25 °C, losses of antioxidant activity between 11.28 and 49.63% were observed.

### 3.3. Carotenoids Bioaccessibility

It is important to assess the release behavior of lycopene from microcapsules over the gastrointestinal tract after ingestion in order to evaluate the potential of wall materials for oral delivery in food or as a supplement for controlled/targeted release in specific zones of the gastrointestinal tract. In vitro release studies of microcapsules loaded with tomato pomace extract were performed under pH and temperature values that simulate the conditions inside the gastrointestinal tract. First, the release profile of the bioactives from the particle samples in each digestive fluid was analyzed separately. Then, the particles produced with inulin were selected to be incorporated into the natural yogurt, since they demonstrated a greater protection in the SGF, and the release rate in simulated gastrointestinal tract was also analyzed.

#### 3.3.1. Microparticles Loaded with Tomato Pomace Extract

**Figure 3:**
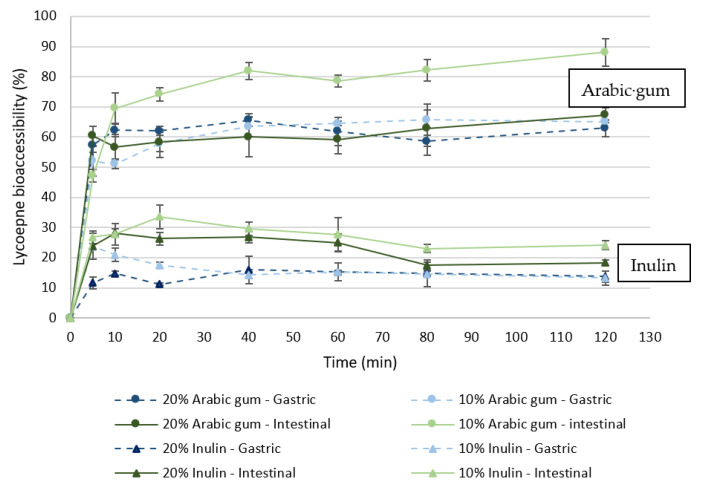
In vitro release profiles of lycopene from microparticles of inulin and arabic gum, under static conditions, in simulated gastric and intestinal fluids.

A sudden release of lycopene from the microparticles of both wall materials (arabic gum and inulin) occurred within the first 20 min in both simulated digestive fluids. After that, the bioactive release was quite slow and a plateau was reached for most of the cases (Figure 3).

The carotenoid molecules that are adsorbed on the surface of the particles as well as those retained near the surface will be released more quickly, being responsible for the initial burst release. Still, the morphology of the particles can also affect the rapid release of the bioactive compounds through the cracks and the pores formed during production of the microcapsules which facilitate and accelerate the release of the core material from the polymeric matrix [33,34,35]. This initial burst release was also observed for the release of microcapsulated β-carotene formed by complex coacervation using casein and tragacanth gum as wall material [36] as well as in the neem seed oil extract release from arabic gum particles [37] and grape marc phenolics release from particles of whey protein and maltodextrin [38].

As shown in Figure 3, the percentage of lycopene released in simulated digestion in both gastric and intestinal fluids was higher for the arabic gum than for inulin particles. At the end of the simulated digestion period, between 63.0 and 65.1% of lycopene was released from the arabic gum particles in simulated gastric fluid, whereas for the inulin particles the release was between 13.3 and 13.8%. Furthermore, a greater release of lycopene was observed in the simulated intestinal fluid. In this fluid, the results showed a lycopene release between 67.3 and 88.1% for arabic gum particles and between 18.3 and 27.6% for inulin particles. Hence, the microencapsulation technique using inulin as the wall material appeared to be effective in protecting the bioactive compounds during the passage through the stomach (simulated gastric fluid). Rutz, et al. [39] also observed that the release profile of the encapsulated material was dependent on the type of wall material. Carotenoids from palm oil were microencapsulated in chitosan/Carboxymethylcellulose (CMC) and chitosan/sodium tripolyphosphate (TPP) by complex coacervation. They observed that the TPP particles released 53 and 67% of the carotenoids under the simulated gastric and intestinal fluids, while the CMC particles showed lower release percentages with 17 and 20% values for the simulated gastric and intestinal fluids, respectively. According to the authors, the CMC wall material promotes lipophilic interactions compared to TPP, and consequently, allowed for greater retention of carotenoids.

**Figure 4:**
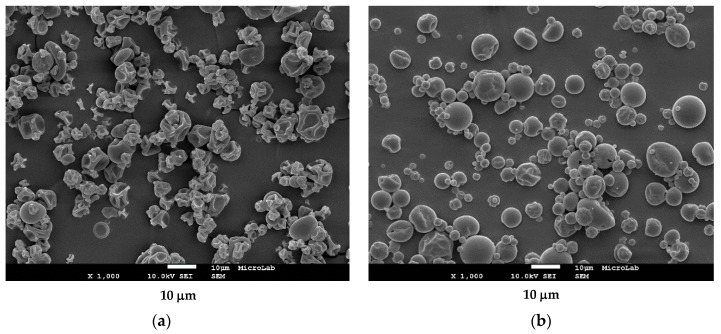
Scanning Electron Microscopy (SEM) images (magnification ×1000) of microparticles loaded with tomato pomace extract in different wall materials. (**a**) Arabic gum particles; (**b**) Inulin particles.

The fact that inulin presents lower release, being an encapsulating agent more protective than arabic gum, can be due to the solubility in aqueous medium of the studied polymers. While inulin has a low solubility in water of 2.5% (*w/v*) at 25 °C, arabic gum has a higher solubility of 50% (*w/v*) [40,41]. Another factor that may influence the release of bioactive microcapsules is their morphology. As shown in Figure 4, the type of wall material used in the production of the particles influenced its morphology. It was observed that for the particles produced with inulin, the surface was smoother while the arabic gum particles showed the formation of teeth and concavities. According to Moreno, Cocero, and Rodríguez-Rojo [38], rough and tooth-forming surfaces can accelerate the release of the microencapsulated compounds due to a larger surface area in contact with the medium as compared to the smoother surfaces.

Other researchers also found the release of bioactives faster on microparticles that had a rough surface. Moreno, Cocero, and Rodríguez-Rojo [38] observed that particles loaded with grape marc phenolics using whey protein isolate as wall material exhibited a wrinkled surface while the maltodextrin particles had a smooth surface. They reported that the phenolic compounds from grape marc present within the whey protein particles showed a lower release rate in the simulated gastrointestinal tract compared to that from the maltodextrin particles. In another study, the release of microencapsulated neem seed oil extract in three different wall materials (polyvinyl alcohol (PVA), arabic gum (AG), and whey protein isolate/maltodextrin) was studied by Sittipummongkol and Pechyen [37]. The authors reported that the particles with the highest release rate of neem seed extract were from the ones that showed more concavities on their surface, in the following order: WPI/MD > GA > PVA.

A significantly lower release of lycopene was observed for the particles produced with the highest wall material concentration (20%), for both arabic gum and inulin, when compared to the results of particles obtained with 10% wall material, during contact with the simulated intestinal fluid (Figure 3). However, during the passage through the simulated gastric fluid, no significant differences were observed. These results indicate that microparticles from 20% wall material demonstrate a better protection of the microencapsulated bioactive compounds in the gastrointestinal system. This difference in release rate of the bioactives found between different concentrations of the same wall material used can be attributed to the size of the particles. The microparticles obtained with the highest polymer concentration are larger when compared to that from lower concentrations, due to the increased viscosity of the solution that induces the formation of larger droplets after spraying. According to Yang, et al. [42], the microparticles produced with lower concentrations of wall material tend to have a more porous surface than microparticles with higher concentrations and consequently, facilitating the release of the encapsulated core material.

#### 3.3.2. Yogurt Enriched with Microparticles

**Figure 5:**
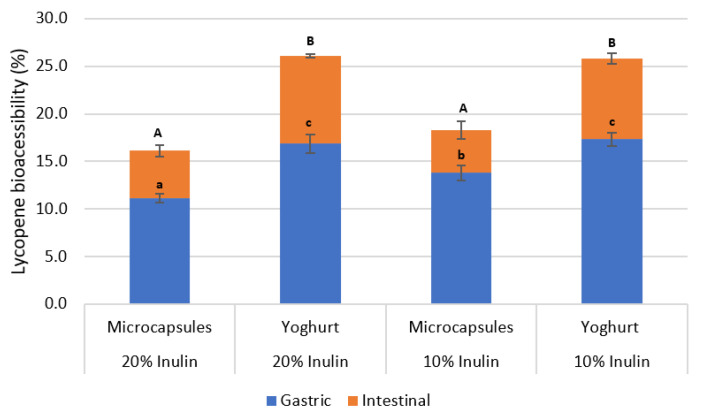
Lycopene release from the microcapsules alone and when incorporated into yoghurt, during a sequential static in vitro digestion: in simulated gastric fluid followed by simulated intestinal fluid. Different letters represent statistically significant differences among treatments (Tukey test *p* < 0.05): uppercase letters (during simulated intestinal fluid); lowercase letters (during simulated gastric fluid).

Microcapsulates produced with 10 and 20% inulin were chosen to be incorporated into liquid natural yogurt. Two parallel release studies were conducted: one using only microparticles and the other using yogurt enriched with microparticles. In the samples using only microparticles, 13.8 ± 0.8 and 11.1 ± 0.5% of the lycopene was released in the simulated gastric fluid from particles produced with 10 and 20% of inulin, respectively (Figure 5). Continuing to the simulated intestinal fluid, the 10 and 20% inulin particles reached the release of 18.3 ± 1.0 and 16.1 ± 0.6% at the end of this simulated stage. When using yogurt enriched with microparticles, the lycopene release increased during the simulated gastric fuid, reaching percentages of 17.4 ± 0.7 and 16.9 ± 0.9% for the concentrations of 10 and 20% of inulin, respectively. In the end of the simulated intestinal stage, a final lycopene release of 25.8 ± 0.5 and 26.1 ± 0.2% was observed for 10 and 20% of inulin, respectively.

As such, the bioaccessibility of lycopene depends both on the digestion stage (gastric or intestinal fluid) and on the food matrix. For both inulin concentrations, a significant increase in lycopene release was observed at each step of simulated gastrointestinal digestion for yogurt with microparticles as compared to particles alone. In particular, comparing only the simulated intestinal stage, the release of lycopene in the yogurt samples was around 85% higher than that of microcapsules alone. Still, no significant differences were observed between the inulin concentrations used after the simulated gastrointestinal digestion.

The improvement in the stability of lycopene in yogurts may be related to the fact that the lycopene molecule has lipophilic characteristics, enhancing its ability to be dissolved in fats and lipids. Therefore, after its release, lycopene was dissolved by the lipid fraction of the yogurt and thus protected from the adverse conditions in the gastrointestinal tract. During simulated digestion only with particles, the lycopene released into the simulated digestive fluid, being hydrophobic, is not readily dissolved, becoming more susceptible to degradation.

Similar results were observed by Rutz, et al. [43] who analyzed the release of carotenoids in yogurt enriched with microparticles loaded with palm oil. The microparticles were produced by complex coacervation followed by lyophilization using chitosan/xanthan as a wall material. The authors observed a greater release of the carotenoids from the particles before incorporation in yogurt. However, some of these released carotenoids were degraded during simulated gastrointestinal digestion, reaching the end of the process with a release of 39.2%. After incorporation into yogurt, no degradation was observed during the simulated gastrointestinal tract, with a total release of 50.1% of microencapsulated carotenoids, thus improving its release and stabilization when added to the food matrix.

The low values in the release of lycopene found in the present work for inulin particles may be also related to its resistance to human digestive enzymes and, consequently, it is not digested in the upper gastrointestinal tract, thus promoting greater protection to the microencapsulated compounds. Inulin is able to reach the colon without being absorbed by the digestive system, and due to its prebiotic characteristics, may serve as a substrate, providing essential energy for endogenous bacteria [44].

### 3.4. Viability of Bacteria from Probiotic Yogurt

**Figure 6:**
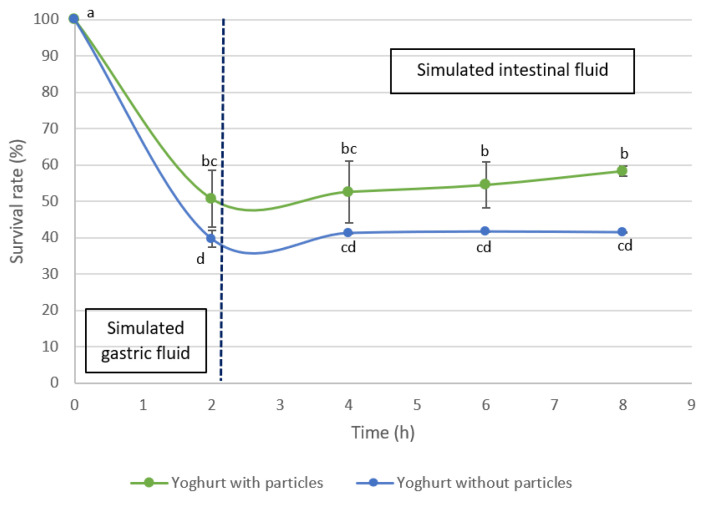
Viability of probiotic bacteria present in yogurt with particles and without particles during simulated gastrointestinal digestion. Different letters represent statistically significant differences between samples during simulated digestion (Tukey test *p* < 0.05).

Based on the release results obtained for the different wall materials of the microcapsules, the particles produced with 20% inulin were chosen for the application in probiotic yogurt (Actimel^®^). The control yogurt (without particles) and yogurt with microencapsulated tomato pomace extract were evaluated for the survival of the probiotics contained in the yogurt during the simulated gastrointestinal tract. The survival rate of the probiotic bacteria of the yogurt with and without microparticles loaded with tomato pomace extract is shown in Figure 6.

It was observed a loss of viability of the probiotic bacteria in yogurt after the simulated gastric digestion for both cases, which was attributed to the acidic conditions present in the gastric fluid (pH = 1.7). There was a reduction of 4.07 and 4.94 log cycles of CFU in the yogurt incorporated with microparticles and in yogurt without microparticles, respectively. This behavior is in agreement with other authors who evaluated the survival of probiotic free cells during the simulated gastric treatment [45,46].

On the other hand, during the simulated intestinal digestion, the survival rate was maintained, and there was a tendency for a higher number of probiotic bacteria present in the yogurt with microparticles. In this stage, the viability of the probiotic bacteria in the yogurt with microparticles was significantly higher when compared that of yogurt without encapsulates. This difference in the viability of the bacteria may be related to the prebiotic characteristics of the inulin, thus serving as a substrate for the probiotic bacteria.

## 4. Conclusions

Storage stability and in vitro release of microencapsulated ethanolic extract from tomato pomace rich in lycopene were influenced by the type and concentration of wall material. Tomato pomace extract, microencapsulated with both inulin and arabic gum as wall materials, was more stable under light and oxygen conditions than the free extract during 27 days of storage. However, between the two different types and concentrations of wall materials studied, inulin used at a concentration of 20% was the one showing the best protection ability of the core material against environmental conditions, in terms of degradation of the lycopene and antioxidant activity. Additionally, during in vitro release studies in simulated gastrointestinal fluids, it was the one that enabled reaching the intestine with a greater concentration of bioactive compounds to be released. Furthermore, after incorporation of the microparticles into yogurts, the bioaccessibility of the microencapsulated lycopene increased during simulated gastrointestinal digestion as compared to the lycopene of the microparticles studied without yogurt. Using inulin as wall material at a concentration of 20% also promoted a higher survival rate of yogurt probiotic bacteria during in vitro gastrointestinal digestion.

## Figures and Tables

**Table 1:** Salt concentrations of simulated gastrointestinal fluids.

Gastric Fluid	Intestinal Fluid
4.8 g/L NaCl	5.0 g/L NaCl
2.2 g/L KCl	0.6 g/L KCl
0.22 g/L CaCl_2_	0.25 g/L CaCl_2_
1.5 g/L NaHCO_3_	

## Data Availability

Not applicable.

## Supplementary Materials

The following supporting information can be downloaded at: https://www.mdpi.com/article/10.3390/bioengineering9070311/s1, Figure S1: HPLC chromatogram obtained with a DAD-3000 diode-array detector, with a wavelength of 475 and 472 nm for lycopene and 440 nm for β-carotene, upon tomato pomace extract analysis.
