# Supplementary material for: Storage Stability and In Vitro Bioaccessibility of Microencapsulated Tomato (Solanum Lycopersicum L.) Pomace Extract
Source: Bioengineering (Basel). 2022 Jul 13;9(7):311. doi: 10.3390/bioengineering9070311 (PMC9312032; doi:10.3390/bioengineering9070311)
Supplement: Supplementary file 1 [file bioengineering-09-00311-s001.zip › bioengineering-1770550-supplementary.pdf]

Supplementary File

Storage stability and in vitro bioaccessibility of microencapsulated tomato (*Solanum lycopersicum* L.) pomace extract

Luiz C. Corrêa-Filho, Diana I. Santos, Luísa Brito, Margarida Moldão-Martins and Vítor D. Alves*

LEAF—Linking Landscape, Environment, Agriculture and Food, Associated Laboratory TERRA, Instituto Superior de Agronomia, Universidade de Lisboa, Tapada da Ajuda, 1349-017 Lisboa, Portugal; lucaalbernaz@gmail.com (L.C.C.-F.); dianaisasantos@isa.ulisboa.pt (D.I.S.); lbrito@isa.ulisboa.pt (L.B.); mmoldao@isa.ulisboa.pt (M.M.-M.)

* Correspondence: vitoralves@isa.ulisboa.pt; Tel.: +351-21-365-3195

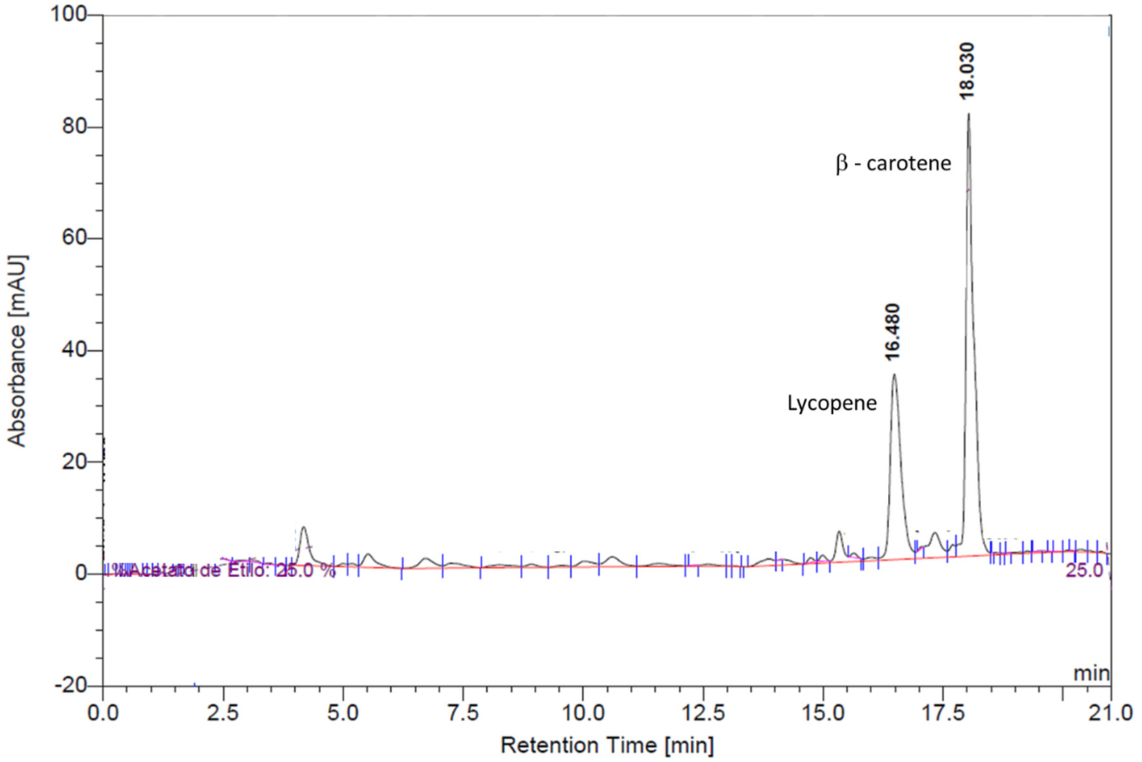

Figure S1. HPLC chromatogram obtained with a DAD-3000 diode-array detector, with a wavelength of 475 and 472 nm for lycopene and 440 nm for β-carotene, upon tomato pomace extract analysis.
